# Potency and selectivity indices of acetone leaf extracts of nine selected South African trees against six opportunistic Enterobacteriaceae isolates from commercial chicken eggs

**DOI:** 10.1186/s12906-017-1597-3

**Published:** 2017-02-02

**Authors:** Ishaku L. Elisha, Alexander R. Jambalang, Francien S. Botha, Elna M Buys, Lyndy J. McGaw, Jacobus N. Eloff

**Affiliations:** 1 Phytomedicine Programme, Department of Paraclinical Sciences, Faculty of Veterinary Science, University of Pretoria, Private Bag X04, Onderstepoort, 0110 Pretoria, South Africa; 2 Permanent address: Drug Development Section, Biochemistry Division, National Veterinary Research Institute, P.M.B. 01 Vom, Plateau State, Nigeria, South Africa; 3 Permanent address: Bacterial Research Division, National Veterinary Research Institute, P.M.B. 01 Vom, Plateau State, Nigeria, South Africa; 4 Department of Food Science, University of Pretoria, Pretoria, South Africa

**Keywords:** Antimicrobial, Plant extracts, Nosocomial, Opportunistic pathogens, Hens’ egg isolates

## Abstract

**Background:**

The rise in antimicrobial resistance in a plethora of nosocomial and opportunistic bacterial pathogens often isolated from commercial eggs, poses a serious public health concern. The existence of these contaminants may also serve as a drawback in the current efforts of improving the well-being of immunocompromised patients. The aim of this study was to determine the efficacy of plant extracts that had good activity on *Escherichia coli* in previous word on pathogens isolated from eggs for possible use in combating pathogens from eggs.

**Methods:**

Acetone leaf extracts of nine trees with good activities against *Escherichia coli* were tested for their in vitro antibacterial activity against six opportunistic bacterial isolates from commercial eggs (*Stenotrophomonas maltophilia, Klebsiella pneumoniae, Salmonella* serotype Typhimurium*, Proteus mirabilis, Enterobacter cloacae* and *Escherichia coli)* using a serial microdilution method with tetrazolium violet as indicator of growth. Cytotoxicity was determined using a tetrazolium-based colorimetric assay against Vero kidney cells, and selectivity index calculated.

**Results:**

The MIC values range of the different extracts against *Stenotrophomonas maltophilia* was 0.08-0.31 mg/ml*, Klebsiella pneumonia* 0.08-0.63 mg/ml, *Salmonella* ser. Typhimurium 0.08-0.63 mg/ml, *Proteus mirabilis* 0.02-1.25 mg/ml, *Enterobacter cloacae* 0.08-0.31 mg/ml and *Escherichia coli* 0.08-0.16 mg/ml respectively. *Escherichia coli* was the most sensitive while *Proteus mirabilis* was most resistant pathogen to the different test extracts, with mean MIC values of 0.08 mg/ml and 0.46 mg/ml respectively. *Cremaspora triflora* extracts had good activity against all the pathogenic egg isolates, with the exception of *Proteus mirabilis. Maesa lanceolata* and *Elaeodendron croceum* had the best total antibacterial activity (TAA), while generally the selectivity index of the extract was low (SI < 1).

**Conclusion:**

The exceptional activity of *C. triflora* extracts suggests that the plant has potential as a therapeutic agent against some members of the Enterobacteriaceae. Further pharmacological investigations may be interesting in the search for new antimicrobial leads.

## Background

Table or hen’s eggs are affordable and rich sources of animal protein. Chicken’s eggs are used as ingredients in a wide variety of foods and refreshments [1, 2]. The rise in consumer knowledge on food safety matters has changed the general public perception of a “good egg” from mere outer shell cleanliness and physical properties to that of microbial integrity [3]. The microbial flora of the hen’s egg before lay is very low [3], after which the shell gets contaminated during the laying process and from surfaces with which it makes contact [4]. As a consequence, enteric pathogens like *Salmonella spp.* and *Escherichia coli* have been isolated from the outer shells of eggs and their internal contents [5]. There are also reports of the isolation of other members of the Enterobacteriaceae, like *Citrobacter spp*., *Enterobacter* spp., *Klebsiella spp., Proteus spp. Providencia spp.* and *Stenotrophomonas maltophilia,* either from whole or cracked eggs. These pathogenic microbes are capable of causing food spoilage, and infectious diseases in consumers when introduced into the food chain [1, 5–7].

There is documented evidence for the development of resistance to antimicrobial agents from an array of pathogens isolated from commercial eggs, triggering serious public health threats and adding pressure to the already overburdened antibiotic resistance crisis [1, 8]. A valid public health concern is that there might be limited active drugs available to treat infected humans, and horizontal transfer of resistant genes between animal and human bacteria may exacerbate the current antimicrobial resistant crisis [9].

One of the measures of combating the rising rates of antimicrobial resistance is the continuous search of new, safe and effective antimicrobials as alternative agents to non-effective ones [10]. Plants have been a source of medicinal agents for thousands of years and a number of contemporary drugs have been developed from natural sources. Many of these isolations were based on the uses of plant extracts in traditional medicine .

In this study, we determined the potency, efficacy and selectivity acetone leaf extracts of nine South African trees with high activity against *Escherichia coli* based on the unpublished PhD thesis of Dr E Pauw [11] against six Gram-negative enteric pathogens isolated from commercial eggs, sold in the Gauteng Province of South Africa. The activity of these extracts have also been examined against *Bacillus anthracis* [12].

## Methods

### Collection of plant material, drying and storage

Fresh leaves from nine South African medicinal plants with excellent activity against *Escherichia coli* were collected from the Lowveld National Botanical Garden, Pretoria National Botanical Garden and University of Pretoria Manie van der Schyff Botanical Garden. Voucher specimens were prepared and sent to the HGWJ Schweickerdt Herbarium of the University of Pretoria for storage and identification. Herewith are the plants with their voucher numbers. *Bolusanthus speciosus* (H. Bolus) Harms (Fabaceae, PRU 120027)*, Calpurnia aurea* (Aiton) Benth ssp. *aurea* (Fabaceae, PRU 120125)*, Maesa lanceolata* Forssk (Maesaceae PRU120125)*, Elaeodendron croceum* (Thunb.) DC (Celastraceae, PRU 120127). *Morus mesozygia* Stapf ex A. Chev (Moraceae, PRU 120128), *Hypericum roeperianum* G.W. Schimp.ex A. Rich. var. *roeperianum,* (Hypericaceae, PRU 120126)*, Cremaspora triflora* (Thonn.) K. Schum (Rubiaceae, PRU 120129)*, Heteromorpha arborescens* (Spreng.) Chan. & Schltdl (Apiaceae, PRU 120026)*, and Pittosporum viridiflorum* Sims (Pittosporaceae, PRU 120025).

### Extraction

Acetone (technical grade, Merck) was used as extractant in the assays, used in a ratio 1:10 ground dried leaf material to extractant. Acetone is the best choice as an extractant mainly due to its ability to extract compounds of a wide range of polarities [13], its non-toxicity to bioassay systems [14] and ease of removal from extracts. Three grams (3.0 g) of each leaf sample were extracted with 30 ml acetone. The resulting suspension was vigorously shaken in 50 ml polyester centrifuge tubes for 5 min, and centrifuged at 4000 x g for 10 min (Hettich Centrifuge, Rotofix 32A, Labotec, Johannesburg, South Africa). The extraction was repeated two more times on the marc and supernatants were decanted into preweighed glass containers after filtering through Whatman No. 1 filter paper and concentrated to dryness under a stream of cold air. The dried extracts were stored at 5 °C in tightly stoppered glass vials until use.

### Bacterial isolation

The WHO standardised culture and isolation methods (gold standard) was used for bacterial isolation. This is dependent on four basic steps namely: (1) Pre-enrichment of samples (shell, albumin and yolk) in non-selective media-buffered peptone water (BPW-Selecta-MEDIA, South Africa). (2) Enrichment of sample-broth in selective media-tetrathionate (Selecta-MEDIA, South Africa). (3) Isolation of sample on selective solid agar media-xylose lysine deoxycholate (XLD-MERCK, South Africa) and (4) Confirmation of presumptive bacterial isolates using biochemical test and molecular techniques like MALDI-TOF and Polymerase Chain Reaction (PCR) [15, 16].

### Test organisms

Microorganisms used in this study represent pathogenic species isolated from commercial hen’s eggs commonly associated with nosocomial and opportunistic infections. The bacteria were isolated from the shell, albumin and yolk, maintained in the Phytomedicine Laboratory at the Faculty of Veterinary Science, University of Pretoria. They consisted of six Gram-negative Enterobacteriaceae strains namely *Stenotrophomonas maltophilia, Escherichia coli, Enterobacter cloacae, Klebsiella pneumoniae, Proteus mirabilis,* and *Salmonella* serotype Typhimurium. All the bacterial strains were subcultured from the original culture, stored at −80 °C on ceramic beads in cryoprotective media (Pro-Lab diagnostics Microbank^@^ 20) and maintained on Müller-Hinton (MH) agar plates at 4 °C.

### Microdilution assay

The bacterial cultures grown overnight were adjusted to McFarland standard 1, equivalent to 1.4 x 10^8^ cfu/ml (*Stenotrophomonas maltophilia*), 3.6 x10^8^ cfu/ml (*Proteus mirabilis*), 3.9 x 10^8^ cfu/ml (*Klebsiella pneumoniae*), 3.8 x 10^8^ cfu/ml (*Escherichia coli*), 3.2 x 10^8^ cfu/ml (*Salmonella serotype* Typhimurium) and 3.6 x 10^8^ cfu/ml (*Enterobacter cloacae*). The two-fold serial dilution microplate method [17] was used to determine the MIC values. Briefly, aliquots (100 μl) of 10 mg/ml solutions dissolved in acetone of the crude extracts were serially diluted with distilled water in 96- well micro titre plates. A 100 μl aliquot of bacterial suspension was added to each well. Sterilised distilled water and acetone were used as negative and solvent control [14], and gentamicin as a positive control against the bacteria. P-iodonitrotetrazolium (INT, 0.2 mg/ml) 40 μl was added after 24 h of incubation of the plant extracts with bacterial cultures at 37 °C and incubated for a further 30 min to 1 h until optimal colour development. The colourless tetrazolium salt acts as an electron acceptor and is reduced to formazan product by biologically active organisms. Tests were carried out in triplicate and each experiment was repeated three times. The MIC was recorded as the lowest concentration of the extract that inhibited bacterial growth [17].

To compare the activity of different plants not only the MIC but also the quantity extracted from the plant should be taken into account. The total activity is calculated by dividing the quantity in mg extracted from one g of plant material by the MIC in mg/ml.

### Cytotoxic activity

The 3-(4,5-dimethylthiazol-2-yl)-2, 5-diphenyltetrazolium bromide (MTT) reduction assay [18] was used to determine the cytotoxicity of the extracts against Vero cells. The method used is described as follows in our previous publication un this journal (12): “Cells were seeded at a density of 1 × 105 cells/ml (100 μl) in 96-well microtitre plates and incubated at 37 °C and 5% CO2 in a humidified environment. After 24 h incubation, 100 μl each of differing extract concentrations were added to the wells containing cells. Doxorubicin was used as a positive control. A suitable blank control with equivalent concentrations of acetone was also included and the plates were incubated for 48 h in a CO2 incubator.

Thereafter, the medium in each well was aspirated from the cells, cells were washed with PBS, and finally 200 μl fresh medium was added to each well. Thirty microliter of MTT (5 mg/ml in PBS) was added to each well and the plates were incubated at 37 °C for 4 h. The medium was aspirated from the wells and DMSO was added to solubilise the formed formazan crystals. The absorbance was measured using a BioTek Synergy microplate reader at 570 nm. The percentage of cell growth inhibition was calculated based on a comparison with untreated cells. The selectivity index values were calculated by dividing cytotoxicity LC50 values by the MIC values in the same units (mg/ml).”

## Results and Discussion

### Activity of the nine acetone leaf extracts against the six egg Enterobacteriaceae isolates

Taking cognisance of the recurring arguments by different investigators on the appropriate cut-off points for defining the strength of antimicrobial activity of plant extracts [19, 20]. Several authors classified plants according to antimicrobial activity [21–23]. This study classified plant extracts with MIC values lower than 0.1 mg/ml as significantly active; those with MIC values >0.1 to 0.625 mg/ml as moderately active, and MIC >0.625 mg/ml with weak or negligible activity.

The total activity of the extracts is a pharmacologically useful measure to compare the efficacy of different plants because it takes into account not only the antimicrobial activities but also the quantities extracted from different plants. The total activity is calculated by dividing the mass in mg obtained from 1 g of plant material by the MIC in mg/ml. The total activity of the extract is expressed in ml/g and is the volume of solvent that can added to the extract obtained from 1 g of plant material that will still inhibit the growth of the specific pathogen [24].

In determining cytotoxicity, plant extracts with LC_50_ > 20 μg/ml were considered relatively non-cytotoxic. Selectivity index values of the extracts were calculated by dividing the cytotoxicity LC_50_ (in mg/ml) by MIC (mg/ml). Plant extracts with SI values >1 imply that the extracts are less toxic to bacteria than to the mammalian cells Plant extracts might have good activity against the test organism but if they are too cytotoxic will not be of any value. When an extract shows consistent low MIC values against different pathogens i.e. there is low selectivity, it is sensible to repeat the assays and recheck the toxicity of the extracts against different cells. If the SI value is low, it could mean that the potency observed against the organisms might be related to a general metabolic toxin in the extract that will affect both the targeted pathogens and the host cells [25]. There is not much incentive for continuing investigation of an extract or isolated compound with a low selectivity index. It should however, be kept in mind that in vivo efficacy and toxicity of extracts or active compounds upon administration to animals or humans may differ substantially from their in vitro properties owing to pharmacokinetic and pharmacodynamical considerations [25]. Infections with Gram-negative bacteria are of imminent concern as they are more difficult to treat and outcome is poor [26].

The tree leaf extracts investigated in this study were selected based on its high activity against *E. coli* [11, 22]. The nine acetone extracts had varying degrees of activity against the six Gram-negative enteropathogenic bacteria isolated from eggs sold in different outlets in the Gauteng province of South Africa. The sensitivity of the pathogens to the plant extracts are discussed below.

### *Stenotrophomonas maltophilia*

*Stenotrophomonas maltophilia* is increasingly recognised as an important nosocomial pathogen. Treatment challenges are due to intrinsic resistance to many antibiotics as well as concerns over toxicity of the drug of choice, co-trimoxazole [27]. *S. maltophilia* is recognised as the cause of skin and soft-tissue, respiratory, bloodstream and prosthetic device infections, especially in immunocompromised and critically ill patients [27]. It is therefore necessary to determine how the various extracts affect *S. maltophilia* isolated from eggs. Given the endemicity of HIV infection in South Africa where the infected persons are often immunocompromised, this is a growing problem in this country [28]. Ingesting improperly cooked foods from contaminated eggs could add to the disease burden, thus contributing to an increase in mortality rates. Within the limits of our literature search, this is the first report of tests carried out using the nine selected South African extracts against *S. maltophilia*. The different extracts tested against *S. maltophilia* had very promising activities with MIC values ranging from 0.08 to 0.31 mg/ml (Table 1). *Cremaspora triflora*, *H. arborescens*, *P. viridiflorum, M. lanceolata and E. croceum* had good activity against *S. maltophilia* with MIC = 0.08 mg/ml.

**Table 1:** Minimum inhibitory concentration (MIC) and total antibacterial activity (TAA) and selectivity index (SI) of the nine selected acetone leaf extracts against *Stenotrophomonas maltophilia*, *Klebsiella sp.* and *Salmonella* serotype Typhimurium (AJ 33)

	*Stenotrophomonas maltophilia*			*Klebsiella pneumoniae*			*Salmonella* ser. Typhimurium (AJ 33)		
Plants	MIC (mg/ml)	TAA (ml/g)	SI	MIC (mg/ml)	TAA (ml/g)	SI	MIC (mg/ml)	TAA (ml/g)	SI
*H. roeperianum*	0.16	749.8	0.41	0.16	749.8	0.41	0.31	387.0	0.21
*C. triflora*	**0.08**	252.1	0.72	**0.08**	252.1	0.72	**0.08**	252.1	0.72
*H. arborescens*	**0.08**	325.4	1.01	0.16	162.7	0.51	0.16	162.7	0.51
*P. viridiflorum*	**0.08**	339.6	0.68	0.16	169.8	0.34	0.16	169.8	0.34
*B. speciosus*	0.31	74.3	0.17	0.16	144.0	0.33	0.63	36.6	0.08
*C. aurea*	0.16	179.0	0.09	**0.08**	357.9	0.17	0.16	179.0	0.09
*M. lanceolata*	**0.08**	1390.4	0.03	0.63	176.6	0^*^	0.16	695.2	0.01
*E. croceum*	**0.08**	1124.6	0.07	0.31	290.2	0.02	0.31	290.2	0.02
*M. mesozygia*	0.16	115.4	0.25	**0.08**	230.8	0.51	0.31	59.6	0.13
Mean	0.13	NA	NA	0.2	NA	NA	0.25	NA	NA
SD	0.07	NA	NA	0.17	NA	NA	0.16	NA	NA
Gentamicin	0.0008	NA	NA	0.0008	NA	NA	0.006	NA	NA

*NA* Not applicable, *AJ* Alexander Jambalang, ^*^ 0.004

The SI and TAA values of the extracts was calculated from the cytotoxicity and percentage yield of the extracts results published in Elisha et al. [12]

Numbers in bold font MIC < 0.1 mg/ml

*Maesa lanceolata* and *E. croceum* extracts had high total antibacterial activity against *S. maltophilia* with TAA = 1390 and 1125 ml/g respectively due to their low MIC values and high percentage extract yield, but they were judged as unsuitable for further investigation due their high cytotoxicity (Table 1). *Maesa lanceolata* extracts have high efficiency against *Bacillus anthracis* and may be a potential disinfectant, especially in poor communities where disinfectants are not readily available or affordable [12]. In the same manner, the potential of the extracts as decontaminants or disinfectants in hatcheries could be explored. *Heteromorpha arborescens* had a selectivity index of 1.01, while the rest of the extracts had SI values lower than 1 (Table 1). Although *H. arborescens* extracts had good a MIC and low cytotoxicity, selecting it for further investigation might be challenging due to low extract yield (Table 1). Furthermore, *H. arborescens* is genotoxic, causing both DNA damage and chromosomal aberrations, and has the potential of causing long-term damage in patients when administered as a medicinal preparation [29].

### *Klebsiella pneumoniae*

*Klebsiella pneumoniae* is the most common Gram-negative bacteria often associated with antibiotic resistant infections [30]. *Klebsiella pneumoniae* is an opportunistic pathogen that infects mostly hospitalised immunocompromised patients causing bacterial pneumonia. The nasopharynx and gastrointestinal tract are its preferred predilection sites [30]. Nosocomial respiratory infections caused by *Klebsiella pneumoniae* (Enterobacteriaceae) have increased in recent years due to the emergence of carbapenemase-producing strains [31]. These bacteria are capable of hydrolysing carbapenems, penicillins, cephalosporins, and aztreonam [31, 32], thus severely challenging antimicrobial therapy [26]. Extracts tested against *K. pneumoniae* isolated from hen’s eggs had MICs ranging from 0.08 mg/ml to 0.63 mg/ml respectively. Acetone extracts of *Cremaspora triflora*, *Calpurnia aurea* and *Morus mesozygia* had good MIC values against *K. pneumoniae* (MICs = 0.08 mg/ml). *Hypericum roeperianum*, *Heteromorpha arborescens*, *Pittosporum viridiflorum*, and *Bolusanthus speciosus* had moderate antibacterial activity against *K. pneumoniae* with MICs of 0.16 mg/ml for all extracts. However, *M. lanceolata* had weak activity with MIC = 0.63 mg/ml. The acetone extracts of *H. roeperianum* and *C. aurea* had total activity values of 750 ml/g and 358 ml/g respectively (Table 1). Other extracts did not meet the benchmark of an acceptable selectivity index, ≥ 1.0, but *C. triflora* had comparatively the highest SI value of 0.72 (Table 1).

### *Salmonella serotype* Typhimurium

The majority of *Salmonella* infections are linked to the consumption of contaminated food of animal origin such as eggs, chicken, and pork. Severe *Salmonella* infections often require antimicrobial therapy to aid in the elimination of the infection. There are concerns over the past three decades regarding the global emergence of multi-drug resistant phenotypes among the *Salmonella* serotypes such as *S.* ser. Typhimurium*, S.* ser. Enteritidis and *S.* ser. Newport [33]. Currently, there are concerns over the emergence of resistance to quinolones, fluoroquinolones or extended-spectrum cephalosporins such as ceftiofur and ceftriaxone. Reports on the occurrence of *Salmonella* isolates resistant to these antibiotics has increased [33]. In view of these negative developments, the acetone extracts of the nine plant species were tested against *S.* ser. Typhimurium isolated from commercial hen’s eggs. The extracts presented varying degrees of activities against the pathogen. The MIC ranged from 0.08 mg/ml to 0.63 mg/ml (Table 1). Acetone extracts of *C. triflora* had good activity against *S.* ser. *Typhimurium* compared to the eight other extracts, with MIC value of 0.08 mg/ml. *Bolusanthus speciosus* had the lowest activity with an MIC of 0.63 mg/ml.

### *Proteus mirabilis*

The antibacterial activity of the extracts against *Proteus mirabilis* ranged from outstanding to poor (0.02 to 1.25 mg/ml). *Proteus mirabilis* had the highest and lowest sensitivity to some of the extracts (Table 2). For example, the extracts of *M. lanceolata* and *E. croceum* had very good activities (MIC = 0.02 mg/ml and 0.08 mg/ml respectively), while the extracts of *M. mesozygia* had the lowest activity with an MIC of 1.25 mg/ml. *Maesa lanceolata* and *E. croceum* had good TAA, with values at 5562 ml/g and 1125 ml/g. All the extracts had low SI values. Cock and van Vuuren [34], reported that methanol and water extracts of *Carpobrotus edulis*, *Lippia javanica*, *Pelargonium viridflorum*, *Ptaeroxylon obliquum*, *Syzygium cordatum* leaf and bark, *Terminalia pruinoides*, *Terminalia sericea*, *Warburgia salutaris* bark and an aqueous extract of *W. salutaris* leaf had some activity *Proteus* inhibitors, with MIC values < 2 mg/ml. Our results are in same order as their results.

**Table 2:** Minimum inhibitory concentration (MIC) and total antibacterial activity (TAA) and selectivity index (SI) of the nine selected acetone leaf extracts against *Proteus mirabilis*, *Enterobacter cloacae* and *Escherichia coli*

	*Proteus mirabilis*			*Enterobacter cloacae*			*Escherichia coli*		
Plants	MIC (mg/ml)	TAA (ml/g)	SI	MIC (mg/ml)	TAA (ml/g)	SI	MIC (mg/ml)	TAA (ml/g)	SI
*H. roeperianum*	0.63	190.4	0.11	0.31	387.0	0.21	**0.08**	1499.6	0.83
*C. triflora*	0.63	32.0	0.09	**0.08**	252.1	0.72	**0.08**	252.1	0.72
*H. arborescens*	0.31	84.0	0.26	0.16	162.7	0.51	**0.08**	325.4	1.01
*P. viridiflorum*	0.31	87.6	0.18	0.16	169.8	0.34	**0.08**	339.6	0.68
*B. speciosus*	0.63	36.6	0.08	**0.08**	287.9	0.66	**0.08**	287.9	0.66
*C. aurea*	0.31	92.7	0.04	**0.08**	357.9	0.17	**0.04**	715.8	0.34
*M. lanceolata*	**0.02**	5561.7	0.12	**0.08**	1390.4	0.03	0.16	695.2	0.01
*E. croceum*	**0.08**	1124.6	0.07	**0.08**	1124.6	0.07	**0.08**	1124.6	0.07
*M. mesozygia*	1.25	14.8	0.03	0.16	115.4	0.25	**0.08**	230.8	0.51
Mean	0.46	NA	NA	0.13	NA	NA	0.08	NA	NA
SD	0.35	NA	NA	0.07	NA	NA	0.03	NA	NA
Gentamicin	0.0008	NA	NA	0.0002	NA	NA	0.006	NA	NA

*NA* Not applicable.

The SI and TAA values of the extracts was calculated from the cytotoxicity and percentage yield of the extracts results published in Elisha et al. [12]

Numbers in bold font MIC < 0.1 mg/ml

### *Enterobacter cloacae*

*Enterobacter* is an increasingly important human pathogen, particularly in the hospital setting. *Enterobacter* accounts for 5 to 11*%* of all nosocomially-transmitted blood, wound, respiratory tract, and urinary tract infections [35]. *Enterobacter* is responsible for between 4 and 12% of all cases of Gram-negative bacteraemia. All age groups are affected. In adults, *Enterobacter* is the third or fourth leading cause of sepsis after *Escherichia coli, Klebsiella* spp., and *Pseudomonas aeruginosa.* All the extracts had reasonable activity against *Enterobacter cloacae*, with MICs ranging between 0.08 and 0.31 mg/ml (Table 2). *Maesa lanceolata* and *E. croceum* had good TAA with values at 1390 ml/g and 1125 ml/g respectively. The susceptibility of *Enterobacter cloacae* has previously been reported by Shiri et al. [35] when tested against ethanol leaf extracts of *Peganum harmal* and *Myrtus communis* respectively.

### *Escherichia coli*

**Fig. 1:**
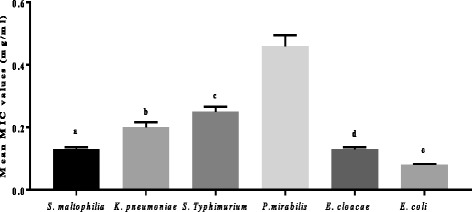
Sensitivity of the six pathogenic egg isolates to the nine selected acetone leaf extracts. Legend: ^a, b, c, d^ and ^e^ = statistically significant difference in the mean MIC of the pathogens, *p* < 0.05

All tested extracts generally, had good activity against *Escherichia coli* egg isolate. MIC values ranged between 0.08 to 0.16 mg/ml. Makhafola and Eloff [36] reported a similar trend when *E. coli* (ATCC 25922) was treated with the extracts from different *Ochna spp. Hypericum roeperianum* and *E. croceum* extracts had a very good TAA, with values of 1500 ml/g and 1125 ml/g respectively. *Heteromorpha arborescens* had the best SI = 1.01 (Table 2). *Maesa lanceolata* and *E. croceum* extracts had poor selectivity index values (Table 2). The mean MIC values of the extracts against the egg isolates ranged from 0.14 mg/ml to 0.34 mg/ml (Tables 1 and 2 and Fig. 1). *Calpurnia aurea* had the lowest mean MIC value of 0.14 ± 0.09 mg/ml, and *M. mesozygia* had the highest mean value of 0.34 ± 0.41 mg/ml (Table 2).

**Fig. 2:**
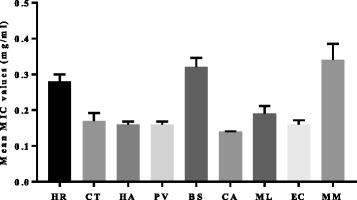
Mean minimal inhibitory concentration of the nine selected acetone leaf extracts tested against six opportunistic bacterial pathogens isolated from commercial eggs. Legend: HR = *Hypericum roeperianum*, CT = *Cremaspora triflora*, HA = *Heteromorpha arborescens*, PV = *Pittosporum viridiflorum*, BS = *Bolusanthus speciosus,* CA = *Calpurnia aurea*, ML = *Maesa lanceolata*, EC = *Elaeodendron croceum*, MM = *Morus mesozygia.* The mean difference of the nine acetone leaf extracts were not statistically significant, *p* > 0.05

In general, extracts of *C. aurea* had good activity against all the tested egg isolates, while extracts of *M. mesozygia* had the lowest activity. *Cremaspora triflora* had good activity against five out of the six pathogens with an MIC of 0.08 mg/ml each, except against *Proteus mirabilis*, were the extract had a moderate activity with (MIC 0.63 mg/ml) (Table 1 and 2). The outstanding activity of *C. triflora* extracts suggests that the plant has potential as a promising therapeutic agent against some members of the Enterobacteriaceae. Further pharmacological investigations might be required in the search for new antimicrobial leads. Also of note are the extracts of *H. arborescens* and *P. viridiflorum* with mean MIC values against all the isolates of 0.16 mg/ml (Fig. 2); they both had similar activity against *P. mirabilis* which also makes them potential candidates for further evaluation.

*Cremaspora triflora* is the best plant extract for investigation because very little work has been carried out on this plant species, thus giving it some novelty. Overall *E. coli* was the most sensitive pathogen to all nine extracts (mean MIC = 0.08 ± 0.08 mg/ml), while *Proteus mirabilis* was the most resistant with mean MIC values of 0.46 ± 035 mg/ml (Table 2 and Fig. 1).

## Conclusions

The extracts had varying degrees of activity against the pathogens isolated from commercial eggs. *Cremaspora triflora* extracts however, had exceptional activities, against all the pathogenic egg isolates except against *Proteus mirabilis.* The promising antibacterial activities and selectivity index of the extracts of *C. triflora* against all test pathogens makes it the good candidate for more detailed pharmacological and biological investigations. If products developed from *C. triflora* extracts are not harmful to humans, it could play a role in enhancing food safety for example by washing the outside of the eggs with an extract before cooking.

## Abbreviations

AJ: Alexander Jambalang.
MIC: Minimum inhibitory concentration
NA: Not applicable
TAA: Total antibacterial activity
